# Supplementary figures and images for: A computational analysis of the oncogenic and anti-tumor immunity role of P4HA3 in human cancers
Source: PLoS Comput Biol. 2024 Nov 6;20(11):e1012284. doi: 10.1371/journal.pcbi.1012284 (PMC11573185; doi:10.1371/journal.pcbi.1012284)

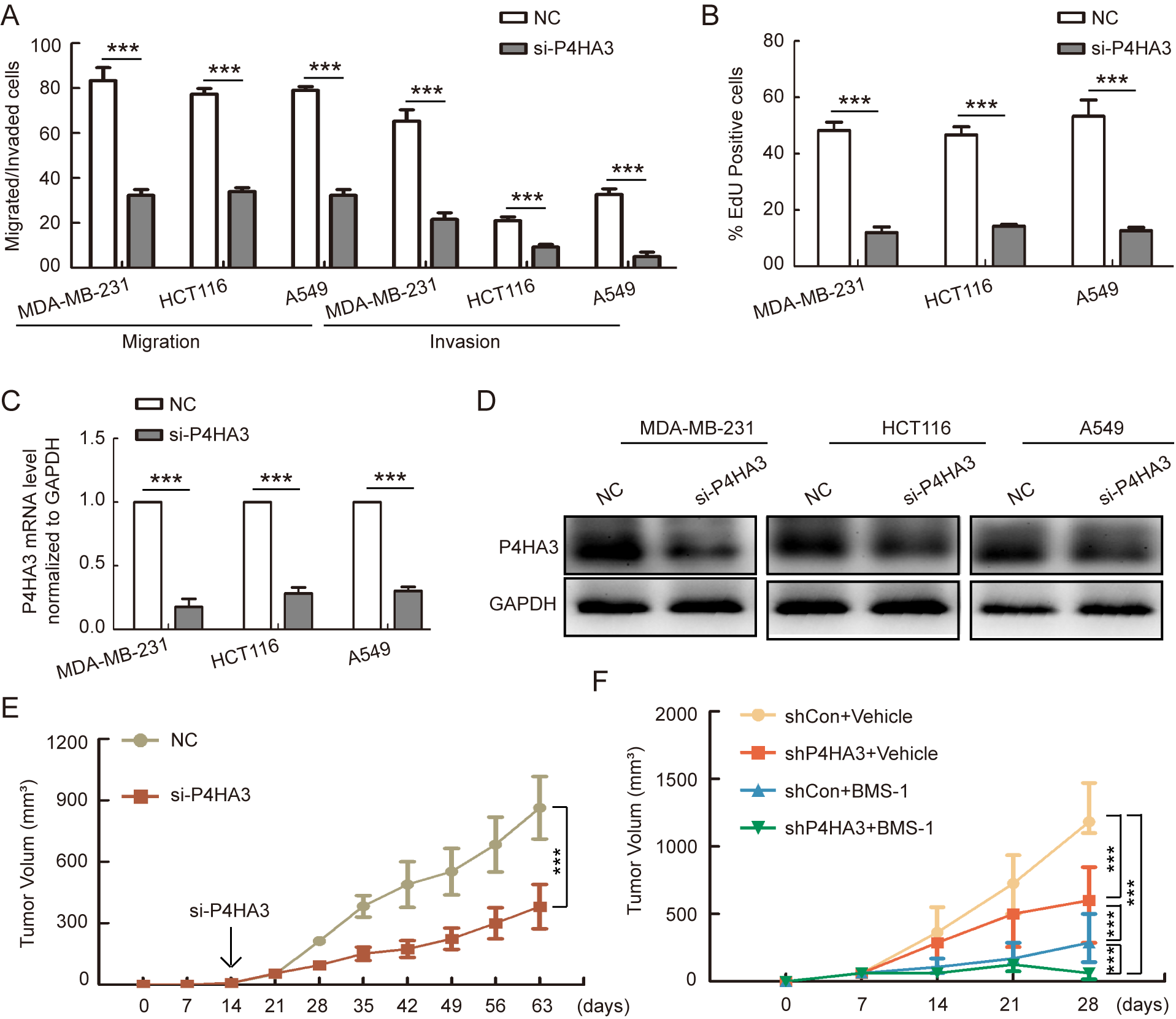

Supplement: S1 Fig — The statistical analysis from Fig 7. (A) P4HA3 deficiency inhibited migration and invasion abilities of MDA-MB-231, HCT116 and A549 cell lines by transwell assay, (***p < 0.001 among the different groups by paired Student´s t tests, mean ± SD). (B) P4HA3 deficiency inhibited proliferation ability of MDA-MB-231, HCT116 and A549 cell lines by EdU assay, (***p < 0.001 among the different groups by paired Student´s t tests, mean ± SD). (C) The knockdown efficiency of P4HA3 in MDA-MB-231, HCT116 and A549 cell lines were detected by using qRT-PCR, (***p < 0.001 among the different groups by paired Student´s t tests, mean ± SD). (D) The knockdown efficiency of P4HA3 in MDA-MB-231, HCT116 and A549 cell lines were detected by using WB. (E) Tumor size in different groups were calculated every 7 days over 2 months. (***p < 0.001 between the NC group and siRNA group by paired Student´s t tests, mean ± SD). (F) Tumor size in different groups were calculated every one week over one month. (***p < 0.001 among the different groups by paired Student´s t tests, mean ± SD). (TIF) [file pcbi.1012284.s001.tif]
