# Supplementary material for: A computational analysis of the oncogenic and anti-tumor immunity role of P4HA3 in human cancers
Source: PLoS Comput Biol. 2024 Nov 6;20(11):e1012284. doi: 10.1371/journal.pcbi.1012284 (PMC11573185; doi:10.1371/journal.pcbi.1012284)
Supplement: Attachment — Submitted filename: Authors response to reviewers tracked change.docx [file pcbi.1012284.s002.docx]

**Response To Reviewers Letter**

Dear Reviewers:

Thanks for the comments concerning our manuscript entitled “A computational analysis of the oncogenic and anti-tumor immunity role of P4HA3 in human cancers” (Manuscript number: PCOMPBIOL-D-24-01089). We appreciate the opportunity to review our firstly revised manuscript from reviewers and be potentially acceptable for publication in PLOS Computational Biology. These comments are valuable and very helpful for improving our manuscript in order to better demonstrate the significance of our research. We have carefully reviewed all comments and revised the text accordingly. The response to the comments in the letter and the minor revised portions of the manuscripts are shown in red fonts. We appreciate the work of the editors and reviewers and hope that our revisions will be met with approval. We will be glad to respond to any further comments that you may have.

Yours sincerely,

Hongyan Huang

Department of Breast Surgery, Zhujiang Hospital, Southern Medical University, Guangzhou 510280, China

Email Address: huangyrc320@smu.edu.cn.

2024-9-14

**Responses to Reviewer 1**

**Comments to the Author**

This paper assesses the role of P4HA3 in cancer development and comprehensive analyses among human cancers, it is a good idea that the authors find P4HA3 deficiency inhibited the proliferation, migration, and invasion abilities of tumor cells, and promoted anti-tumor immunotherapy of PD-1/PD-L1 inhibitors. I think the paper is interesting. However, what is the application of P4HA3 in the clinical tumor gene panel (such as the MSK-MPACT panel)?

**Minor points:**

1-what is the application of P4HA3 in the clinical tumor gene panel (such as the MSK-MPACT panel)?

**Response:** We thank the Reviewer for carefully evaluating our work, and for commenting that this manuscript is interesting. Here, we investigated the relationship between P4HA3 expression and anti-tumor immunity and its prognostic value in pan-cancer. We further found that P4HA3 deficiency inhibited the proliferation, migration, and invasion abilities of tumor cells, and promoted anti-tumor immunotherapy of PD-1/PD-L1 inhibitor, suggesting our study has potential clinical application prospects. Recently, tumor next-generation sequencing has been widely used in clinical gene detection, such as the MSK-MPACT panel and Myriad myChoice CDx. These panels are mainly based on the detection of genetic mutations. There are three versions of the MSK-MPACT panel, each containing a different number of genes: Version 1 has 341 genes, Version 2 has 410 genes, and Version 3 has 468 genes. However, none of the three versions of the MSK-MPACT panel contained the P4HA3. This may be due to the low frequency of P4HA3 mutations in tumors.

In our study, the pan-cancer analysis of P4HA3 provides a comprehensive understanding of its oncogenic and prognosis role in different cancers, P4HA3 abnormal expression could be a useful biomarker for predicting the effectiveness of immunotherapy in cancer patients.

**Responses to Reviewer 2**

**Comments to the Author**

Huang et al. systematically describe the oncogenic and anti-tumor immunity of P4HA3 in human cancers, which P4HA3 is generally over-expressed in human cancers to drive proliferative and metastasis properties. The current manuscript focuses on the role of P4HA3 in various of cancers and anti-tumor immunotherapy of PD-1/PD-L1 inhibitor in triple-negative breast cancer. The investigators intensively completed P4HA3 expression by using different databases including TIMER2.0, GTEx, GEPIA2.0 and TCGA. Genetic and DNA methylation alterations, survival analysis and proteins co-expression analysis of P4HA3 in cBio Cancer Genomics Portal, TCGA, GSCA and TIMER2.0. The correlation between P4HA3 expression and immune infiltration was analyzed by TIDE, XCELL, MCPCOUNTER, and EPIC. Besides above computational analyses, they performed some molecular experiments to confirm the functional role of P4HA3 in different tumors. More importantly, they again verified anti-tumor immunity of P4HA3 by many clinical data. Using these data, the authors conclude that P4HA3 abnormal expression could be a useful biomarker for predicting the effectiveness of immunotherapy in cancer patients. This study has a strong innovation, but, i found many small problems to be resolved before publication:

**Minor points:**
1- Three are many grammatical errors throughout the manuscript.

**Response:** We are very sorry for our grammatical errors and incorrect writing and we have checked all the writing in our paper carefully again. Meanwhile, in order to improve the writing of this manuscript, this paper was polished by highly qualified native speaking editors. We have carefully revised the manuscript and marked the changes in red.

2- Please number your figure and provide the figure legends as it will make it easier to understand your work.

**Response:** We apologize for our negligence and the lack of figure legends. We have added the figures and figure legends in the revised manuscript.

3- The mousseline is incorrectly written as NOSCID instead of NOD-SCID mice.

**Response:** We appreciate this considerate comment, and we apologize for our careless about the incorrect description. We changed NOD-SCID into NOSCID in the whole paper.

4- Please provide the patient information of PDX model.

Response: We apologize for our negligence and the lack of PDX´s patient information, and we are very appreciate this valuable comment. We have added the information (the tumor size was about 3.5 cm, the patient age´s age was 48 years old) in the revised manuscript.

**Responses to Reviewer 3**

**Comments to the Author**

This manuscript is a work of tour-de-force that provides a comprehensive understanding of the P4HA3 oncogenic and prognosis role in different cancers. Their main findings are the unappreciated antitumor therapeutic effect of P4HA3, which might be a useful biomarker for predicting the effectiveness of immunotherapy in cancer patients. Overall, this is solid work that could benefit from some minor improvements.

**Response:** It is with great pleasure to note that the Reviewer considers this is a work of tour-de-force!

**Minor points:**

1-In functional experiments, the authors focused on breast cancer. However, the expression of P4HA3 was not associated with overall survival in the TCGA breast cancer patients. How does P4HA3 expression affect the survival of breast cancer patients in the other dataset?

Response: We would like to thank the reviewers for their comments. We then used another large sample database (Kaplan Meier-plotter) to analyze the effect of P4HA3 gene expression on breast cancer patients. Notably, Elevated P4HA3 expression was correlated with poor OS in breast cancer patients (Figure R1). This is consistent with our previous findings, suggesting that P4HA3 is an oncogene in breast cancer. Figure R1 was showed in below:

**Figure R1. The effect of P4HA3 gene expression on overall survival of breast cancer.** Kaplan-Meier survival analysis of 2976 patients with breast cancer based on P4HA3 gene expression via Kaplan-Meier Plotter (<https://kmplot.com/analysis/index.php?p=background>).

2-The banding of P4HA3 in Figure 7H is somewhat misunderstood: the protein expression of P4HA3 seems to be higher in the P4HA3 knockdown group (shP4HA3) than in the control group. Please check the data carefully.

**Response:** We apologize for our negligence and appreciate this valuable comment, this is a mistake in our picture integration. We have corrected it in the revised manuscript. We also examined the other Figures carefully. was showed in below:
